# Influence of Antiphospholipid Antibody-Associated Thrombophilia on the Risk of Preterm Birth: A Systematic Review

**DOI:** 10.3390/jcm12165316

**Published:** 2023-08-15

**Authors:** Olivera Iordache, Doru Mihai Anastasiu, Manaswini Kakarla, Ayesha Ali, Felix Bratosin, Radu Neamtu, Catalin Dumitru, Flavius Olaru, Izabella Erdelean, Angelica Gherman, Cecilia Roberta Avram, Lavinia Stelea

**Affiliations:** 1 Department of Obstetrics and Gynecology, “Victor Babes” University of Medicine and Pharmacy Timisoara, 300041 Timisoara, Romania; gindacolivera@yahoo.com (O.I.); doru_anastasiu@yahoo.com (D.M.A.); radu.neamtu@umft.ro (R.N.); olaru.flavius@umft.ro (F.O.); erdelean.izabella@gmail.com (I.E.); stelea.lavinia@umft.ro (L.S.); 2 Doctoral School, “Victor Babes” University of Medicine and Pharmacy, Eftimie Murgu Square 2, 300041 Timisoara, Romania; felix.bratosin@umft.ro; 3 Kamineni Institute of Medical Sciences, School of Medicine, Hyderabad 500001, Telangana, India; manaswinikakarla@gmail.com; 4 Bhaskar Medical College, Amdapur Road 156-162, Hyderabad 500075, Telangana, India; ayeshaaliflossy@gmail.com; 5 Department of Infectious Diseases, “Victor Babes” University of Medicine and Pharmacy, Eftimie Murgu Square 2, 300041 Timisoara, Romania; 6 Research Center for Medical Communication, “Victor Babes” University of Medicine and Pharmacy, Eftimie Murgu Square 2, 300041 Timisoara, Romania; angelaeden30@yahoo.com; 7 Department of Residential Training and Post-University Training, “Vasile Goldis” Western University, Liviu Rebreanu Street 86, 310414 Arad, Romania; avram.cecilia@uvvg.ro

**Keywords:** thrombophilia, antiphospholipid syndrome, pregnancy, pregnancy complications, preterm birth

## Abstract

Antiphospholipid antibody (aPL)-associated thrombophilia has been implicated in various adverse pregnancy outcomes, including preterm birth and impaired fetal development. This systematic review aimed to elucidate the relationship between aPL-associated thrombophilia and these outcomes, as well as to identify potential modifiers of this relationship such as maternal age, coexisting maternal medical conditions, type of aPL antibodies involved, and the timing of thrombophilia diagnosis during gestation. We conducted a comprehensive literature search in PubMed, Web of Science, Cochrane, and Scopus in May 2023, covering literature published within the last 10 years. Eight articles, involving 2935 patients, were eligible for inclusion in the review. Single aCL was the most common type of aPL found in patients, with rates up to 61.0% in some studies, followed by single LA and single ab2GPI. Multiple aPL antibody positivity was found to be associated with a higher risk of preterm birth, with odds ratios ranging from 1.29 to 9.61. Patient characteristics and previous pregnancy history varied significantly across the studies. Risk factors such as diabetes mellitus, thrombosis, and systemic lupus erythematosus were also variable across the studies, but presence of these risk factors did not consistently affect the risk of preterm birth. Furthermore, although a triple positive aPL test was the most important risk factor for preterm birth, it was observed that thrombophilia treatment during pregnancy significantly reduced the risk by 2.44 times (95% CI = 1.18–6.20). This review supports the evidence for aPL-associated thrombophilia being a significant contributor to preterm birth and fetal developmental abnormalities. Further research is required to investigate the exact mechanisms and to determine the best clinical management for patients with aPL-associated thrombophilia during pregnancy.

## 1. Introduction

Preterm birth, defined as birth before 37 weeks of gestation, is a significant global health concern [1,2]. It is associated with considerable morbidity and mortality, contributing to 75% of perinatal mortality and more than half of long-term morbidity [3]. Multiple factors contribute to preterm birth, including maternal health conditions, lifestyle, genetic predisposition, and immunological factors. One such immunological factor under scrutiny is antiphospholipid syndrome (APS) [4,5,6].

APS is an autoimmune disorder characterized by thrombosis and pregnancy-related complications, including recurrent miscarriage, preeclampsia, and preterm birth [7]. At the background of this condition lies the persistent presence of antiphospholipid antibodies (aPL)–lupus anticoagulant, anticardiolipin antibodies, and anti-β2-glycoprotein I antibodies [8]. These antibodies promote a pro-thrombotic state by interfering with the coagulation cascade and endothelial function, thereby increasing the risk of vascular complications [9].

In pregnancy, APS is associated with an increased risk of adverse obstetric outcomes. The immunological responses and inflammatory state induced by aPL can interfere with placental function, leading to fetal growth restriction, stillbirth, preeclampsia, and preterm birth [10,11]. Despite advances in understanding the role of aPL in pregnancy complications, the mechanistic underpinnings connecting aPL-associated thrombophilia and preterm birth remain only partially understood [12,13].

Previous studies investigating the relationship between aPL-associated thrombophilia and preterm birth have reported mixed results, highlighting the need for a comprehensive review of the existing literature [14,15]. Further, considering the potential impact on clinical practice—specifically in the prevention and management of preterm birth in women with APS—a systematic investigation of this issue is clearly warranted.

The primary hypothesis of this study is that aPL-associated thrombophilia significantly increases the risk of preterm birth. We hypothesize that this risk may be modulated by several factors, such as the specific type of aPL present, the patient’s history of thrombotic events, and co-existing autoimmune disorders. Additionally, it was assumed that the effect of aPL-associated thrombophilia on preterm birth may be mediated by complications such as preeclampsia and intrauterine growth restriction. By addressing these research questions, this systematic review aims to improve our understanding of APS’s impact on preterm birth, providing valuable insights for clinicians and researchers alike. Therefore, we conducted a systematic review focusing on the influence of aPL-associated thrombophilia on the risk of preterm birth, aiming to quantify the risk of preterm birth associated with aPL-associated thrombophilia. Secondly, we aimed to elucidate the effect of various clinical and demographic factors on this risk. Lastly, we also aimed to identify gaps in the current knowledge base, thus providing direction for future research.

## 2. Materials and Methods

### 2.1. Review Protocol

This systematic review was conducted in May 2023 and involved searching four electronic databases: PubMed, Web of Science, Cochrane, and Scopus. The review included literature published within the last 10 years. The search strategy utilized Medical Subject Headings (MeSH) keywords, such as “thrombophilia”, “antiphospholipid antibodies”, “anticardiolipin antibodies”, “lupus anticoagulant”, “anti-β2-glycoprotein”, “aPL”, “intrauterine growth restriction”, “preterm birth”, “preterm delivery”, “prematurity”, “IUGR”, “fetal development”, and “adverse fetal outcomes”. The search was limited to English-language journal articles.

In adherence to the Preferred Reporting Items for Systematic Reviews and Meta-Analyses (PRISMA) criteria [16] and the International Prospective Register of Systematic Reviews (PROSPERO) guidelines [17], a structured and systematic search strategy was employed to identify relevant scientific papers examining the influence of aPL-associated thrombophilia on IUGR and fetal development. This systematic review was registered on the Open Science Framework (OSF) platform [18].

The primary objective of this systematic review was to comprehensively explore and address research questions that assess the relationship between aPL-associated thrombophilia and preterm birth, as well as overall fetal development. The main research question was aimed at determining the impact of aPL-associated thrombophilia on the incidence of premature births and fetal developmental abnormalities. The review also sought to identify any significant modifiers that might influence this relationship. These modifiers included maternal age, coexisting maternal medical conditions, type of aPL antibodies involved, and the gestational age at which thrombophilia was diagnosed.

### 2.2. Selection Process

Primary sources of information for the compiled material encompassed the text, tables, figures, and available data within the articles. The selection process began with study exclusion based on title and abstract. Duplicate studies were excluded during the screening part. This was followed by a thorough evaluation by two independent researchers of each abstract to assess its relevance to the research questions. Subsequently, a comprehensive review of the entire text was conducted for the remaining articles to ensure that they met the inclusion criteria. An in-depth analysis of the reference lists of the gathered papers was performed by two independent researchers, to identify any pertinent literature that may have been overlooked during the initial search, thereby enhancing the comprehensiveness of this systematic review.

The inclusion criteria for studies in the systematic review were as follows: (1) the study should examine the association between aPL-associated thrombophilia and preterm birth or fetal development; (2) the study must have specifically measured the presence of aPLs; (3) the research must have detailed fetal outcomes; and (4) the study cohort must include pregnant women diagnosed with aPL-associated thrombophilia. Conversely, the exclusion criteria were: (1) studies where aPLs were not measured; (2) studies lacking relevant data on patients’ characteristics and medical history; (3) articles where preterm birth or fetal development were not mentioned; (4) studies where the preterm risk was not evaluated (hazard ratio, risk ratio, odds ratio); (5) antiplatelet therapy being used in the study groups; and (6) other exclusions were made for case reports, literature reviews, meta-analyses, letters to editors, and brief communications.

The final analysis included a broad set of variables, encompassing both study characteristics and key findings. The study characteristics evaluated included the study number and first author’s name, the geographical location of the study, the year the study was conducted, the research design used, and a thorough assessment of the study’s quality. Key findings, such as the presence of aPLs, incidence and severity of prematurity, and measures of fetal development, were extracted from each study.

### 2.3. Data Extraction and Quality Assessment

**Figure 1:**
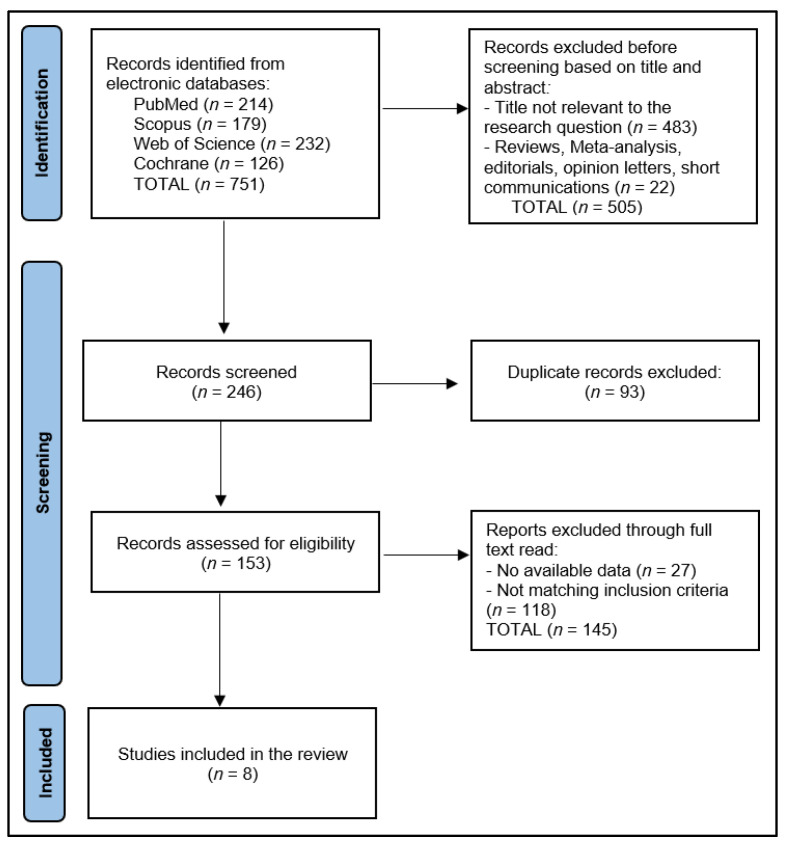
PRISMA flow diagram.

The initial exploration yielded a total of 751 studies, of which 93 were recognized as duplicates. After eliminating 505 papers based on their title and abstracts, we scrutinized 153 full-text articles for relevance. Ultimately, eight articles were eligible for inclusion in the systematic review, as shown in Figure 1. Utilizing the Study Quality Assessment Tools from the National Heart, Lung, and Blood Institute (NHLBI) [19], two investigators independently appraised the published works and recorded their conclusions.

### 2.4. Assessment of Publication Bias and Study Quality

Publication bias was examined by creating a funnel plot, where the standard error of the log odds ratio was plotted against its corresponding log odds ratio. The symmetry of the plot was visually examined and further assessed using Egger’s regression test, with a *p*-value < 0.05 indicating significant publication bias. A sensitivity analysis was also conducted by removing one study at a time and recalculating the pooled odds ratios to evaluate the robustness of the results and to examine the impact of individual studies on the overall effect size.

The Quality Assessment Tool for Observational Cohort and Cross-Sectional Studies was used to evaluate the included studies [20]. Each question within the tool received a score of 1 for “Yes” responses or 0 for “No” or “Other” responses, to determine the final performance score. Studies with scores from 0 to 4 were labeled as fair quality, those scoring between 5 and 9 were labeled as good quality, and those with a score of 10 or above were deemed excellent quality.

## 3. Results

### 3.1. Study Characterisitcs

**Figure 2:**
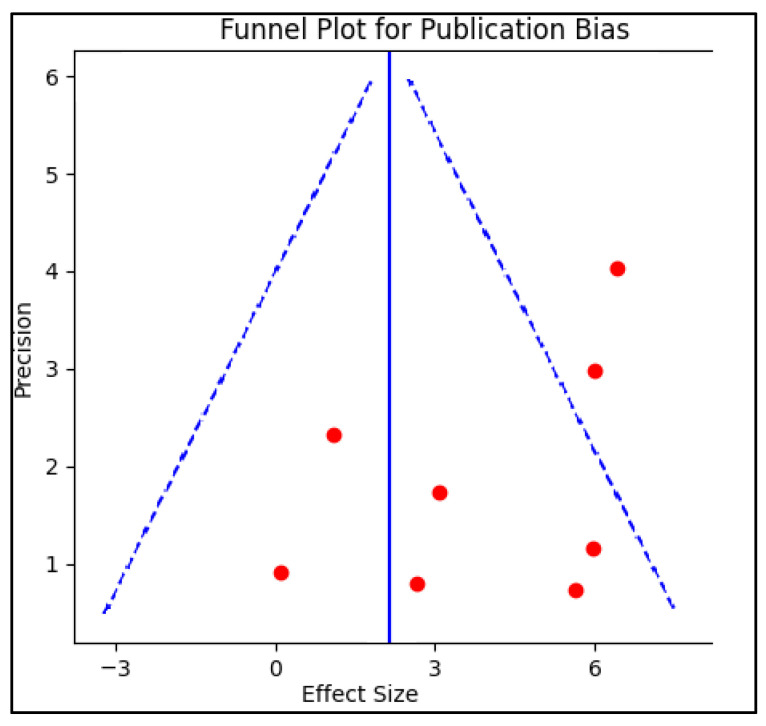
Funnel plot for publication bias.

In the review, eight studies published between 2016 and 2022 were assessed that investigated the influence of antiphospholipid antibody-associated thrombophilia on the risk of preterm birth, and were evaluated for publication bias, as presented in Figure 2. Studies varied by location, design, and quality but demonstrated an overarching agreement in the identified influence of antiphospholipid antibodies on preterm birth risk.

Among the included studies [21,22,23,24,25,26,27,28], two different study designs were utilized: cohort studies, either retrospective or prospective, and case–control studies. Specifically, two studies followed a prospective cohort design [22,24], five studies adopted a retrospective cohort design [21,25,26,27], and one study was a case–control study [28]. The prospective cohort studies, carried out in France [22] and Egypt [24], were noted for their design strength, with the French study being described as excellent in quality, although the Egyptian study was ranked as fair, as seen in Table 1.

By comparison, the five retrospective cohort studies, conducted in various countries including Italy [21], Japan [25], Israel [26], and Romania [27], revealed variable study quality, ranging from fair to good. It is important to note that despite differences in quality, the retrospective studies generally agreed in their findings, indicating a consistent association between antiphospholipid antibodies and preterm birth. The case–control study conducted in China [28] was assessed to be of fair quality. While the study design of a case–control study is different from that of cohort studies, it nevertheless reinforced the findings of the other studies regarding the influence of antiphospholipid antibodies on preterm birth risk.

### 3.2. Patients Characterisitcs

Patient characteristics were gleaned from eight diverse studies assessing the impact of antiphospholipid antibody-associated thrombophilia on preterm birth risk. This covered an aggregate of 2935 patients, with individual study sizes ranging from a low of 81 [25] to a high of 1000 participants [23]. The average age of participants varied across studies, from as low as 27.8 years [21] to an elevated percentage of 42.9% of participants being 35 years or older [27]. Body mass index (BMI), an important factor in evaluating overall health, also exhibited variability across studies, with available data ranging from an average of 19.2% of participants having a BMI over 25 [27] to a mean BMI of 26.0 kg/m^2^ [22]. However, it is important to note that BMI data were not reported in three of the studies [24,25,28].

**Figure 3:**
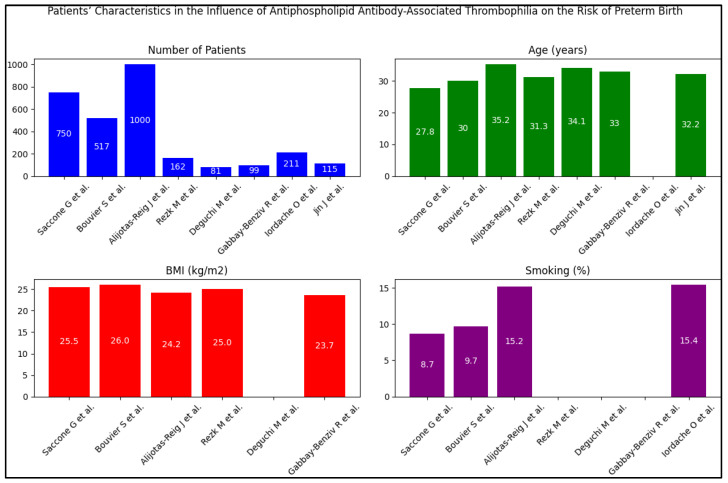
Patients’ characteristics [21,22,23,24,25,26,27,28].

In terms of ethnicity, where reported, a predominant proportion of patients were identified as Caucasian, ranging from 72.5% [23] to 94.6% [22]. Other ethnicities, such as African-American and Asian, were represented to a lesser extent in the participant pool. However, ethnicity was not reported in three of the studies [24,25,27]. Smoking status, a recognized risk factor for preterm birth, was reported in four studies, with the prevalence ranging from 8.7% [21] to 15.4% [27]. Unfortunately, data on smoking were not reported in the remaining studies [24,25,26,28], as described in Table 2 and Figure 3.

### 3.3. Pregnancy and Patients’ Outcomes

**Figure 4:**
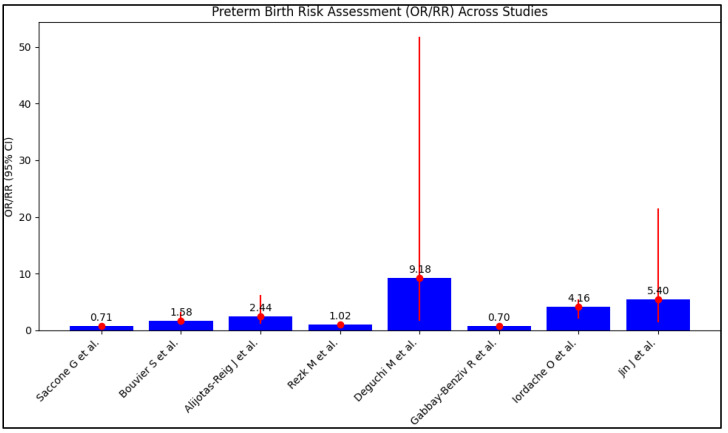
Preterm birth risk assessment across studies [21,22,23,24,25,26,27,28].

Patient’s previous pregnancy history differed significantly across the included studies. The proportion of patients with a history of live birth ranged from a low of 11.8% [27] to a high of 82.7% [25]. In relation to previous pregnancy loss, the percentage of patients who experienced fetal loss before 10 weeks gestation also varied significantly, with a rate as high as 74.4% reported in the study by Iordache O et al. [27], in contrast to lower proportions in other studies. The history of risk factors, including diabetes mellitus (DM), thrombosis, systemic lupus erythematosus (SLE), and fetal loss, revealed variability across the studies. DM prevalence ranged from 1.8% [23] to 11.1% [26], while the reported prevalence of thrombosis fluctuated significantly from 0.0% [22] to 33.3% [26]. The rates of fetal loss before 10 weeks varied from 18.2% [26] to 74.4% [27]. Notably, two studies reported a high rate of thrombosis, at 29.6% [25] and 33.3% [26] respectively, and the latter study also noted a 17.2% prevalence of SLE, as observed in Table 3 and Figure 4.

Concerning the presence of APL antibodies, the distribution of lupus anticoagulant (LA), anticardiolipin (aCL), and anti-β2 glycoprotein I (ab2GPI) antibodies demonstrated wide-ranging results. While some studies showed higher percentages of single LA, aCL, or ab2GPI positivity, others documented a notable proportion of patients with double or triple positivity. This indicates the diverse characteristics of the antiphospholipid antibody-associated thrombophilia in the included populations. The assessment of preterm birth risk associated with the presence of APL antibodies was diverse across the studies. While some studies found that single positivity for APL antibodies was associated with a lower risk of preterm birth compared to multiple positivity [21,26], others showed an increased risk associated with double or triple positivity [25,28]. The study by Iordache O et al. [27] provided an intriguing insight into the potential risk of pregnancy loss associated with APL antibodies, presenting higher odds ratios for both first and second trimester pregnancy loss.

## 4. Discussion

### 4.1. Current Findings

The current systematic review aimed to investigate the impact of antiphospholipid antibody-associated thrombophilia on the risk of preterm birth and overall fetal development. We found overarching agreement across these studies, indicating a consistent association between aPL antibodies and an increased risk of preterm birth. There was a considerable variability in the prevalence of single and multiple aPL antibody positivity across the studies, illustrating the diverse nature of the antiphospholipid antibody-associated thrombophilia in the respective populations. While a number of studies indicated higher percentages of single lupus anticoagulant (LA), anticardiolipin (aCL), or anti-β2 glycoprotein I (ab2GPI) positivity, others noted a substantial proportion of patients with double or triple positivity. This variability could potentially reflect differences in the studied populations, diagnostic criteria, or clinical management.

Patient characteristics, such as age, BMI, ethnicity, and smoking status, varied considerably across the studies and could potentially modify the relationship between aPL-associated thrombophilia and preterm birth. Age and BMI, which have been linked to obstetric complications, varied significantly between studies. Similarly, ethnicity, known to influence pregnancy outcomes, was predominantly Caucasian, but this varied between studies. Lastly, smoking status, a known risk factor for preterm birth, also exhibited significant differences across studies.

The studies included in our review had a wide range of participants, varying from 81 to 1000, making it one of the most comprehensive reviews on this topic to date. Despite the diversity in study size, design, and quality, our findings consistently suggested an increased risk of preterm birth in the presence of aPL antibodies. Moreover, the studies were conducted in various geographical locations, encompassing both Western and Eastern populations, which enhances the generalizability of our findings.

Our findings suggest an increased risk of preterm birth in the presence of aPL antibodies, with some variations noted, where Rezk et al.’s study [24] found a lower risk of preterm birth in patients with triple positivity for aPL antibodies, (OR = 1.29), compared with Deguchi’s study [25], which identified a much higher risk that was 9.61 times higher. Moreover, four studies compared the presence of single aPL antibodies to those with multiple positivity [21,25,26,28]. It is worth noting that, despite these differences, seven of the included studies consistently pointed towards an increased risk of preterm birth in the presence of aPL antibodies, thus reinforcing the primary hypothesis of our review. The only exception was the study performed by Bouvier et al. [22], who identified an insignificant risk for preterm birth in the presence of aCL and ab2GPI antibodies. However, the concerning point regarding this study is the large sample size of 517 patients that provided sufficient statistical power.

The significance of specific aPL antibodies on preterm birth risk varied between studies. For example, a study by Bouvier S et al. [22] noted that the presence of aCL and ab2GPI antibodies increased the risk of preterm birth, while Rezk M et al. [24] pointed out that triple positivity increased the risk compared to ab2GPI antibodies. These findings suggest that the types of aPL antibodies might influence the risk of preterm birth, but more research is needed to better understand these relationships.

Besides the risk of preterm birth, other large studies investigated the influence of aPL antibodies in other pregnancy conditions such as low birth weight or being born small for gestational age, which can be correlated with premature labor. Concordant with preceding reviews was the association among prior conditions such as thrombosis and triple aPL positivity with diverse unfavorable pregnancy outcomes (small for gestational age, preeclampsia, or neonatal mortality) [29,30]. Regarding specific antiphospholipid antibodies, another review [31] suggested that lupus anticoagulant (LA) is the primary predictor of negative pregnancy outcomes, although one study indicated that there is conflicting evidence concerning the predictive value of LA [30]. One meta-analysis discovered significant correlations between LA and several adverse pregnancy outcomes with minor levels of heterogeneity [32]. However, the associations between the presence of ab2GP1 or aCL antibodies and negative pregnancy outcomes were less frequently reported in the included studies. As a result, available data were either too sparse for aggregation or led to substantial heterogeneity. However, the findings cannot be entirely validated due to the increased possibility of a simultaneous autoimmune disease associated with these patients who had positive aPL. Only four studies [33,34,35,36] examined the presence of simultaneous autoimmune disease in relation to the studied adverse pregnancy outcomes, and none of them yielded any significant results, either clinically or statistically.

Moreover, the nature of the antibodies or association of antibodies that were identified in other studies as negative predictors for low birth weight, neonatal mortality, and preterm birth remained indeterminate. The EULAR [37] categorized LA and triple aPL positivity as high-risk aPL profiles for both thrombotic and adverse obstetric events. These antibodies could hypothetically disrupt placentation, thus predisposing patients to fetal loss, placental insufficiency, and preeclampsia [32]. A high-risk subgroup for pregnancy complications, identified by this meta-analysis, comprised patients with thrombotic APS.

It is noteworthy that, while placental infarction is more prevalent in aPL-positive women compared to those without aPL, this is not the universal feature in cases of fetal loss [38]. In addition, thromboses of the spiral artery, placental vessel or intervillous are not typically found in the placentas of aPL-positive women [38]. Contemporary evidence suggests that obstetric morbidity is mainly due to placental inflammation, inhibition of trophoblast proliferation and function, and complement activation [39]. Thrombotic APS is also believed to be significantly influenced by immune cell and complement activation. Hence, the hypothesis could be that thrombosis does not directly cause adverse obstetric events, but rather, both manifestations of APS follow a common pathway. This line of thought could also elucidate why previous thrombosis is among the strongest predictors of pregnancy complications.

To evaluate the risk factors for preterm birth, our review also considered the history of previous miscarriages and thrombosis across the included studies. While a significant variation was observed in the rates of previous pregnancy loss and thrombosis among the subjects, a direct statistical relationship between these factors and preterm birth was not established in our analysis. Specifically, previous pregnancy loss ranged from 18.2% to 74.4% across studies, and thrombosis prevalence varied from 0.0% to 33.3%. Although these factors were comprehensively described, they were not quantified in relation to preterm birth risk. Our findings suggest that these factors may play a role in understanding the complex landscape of preterm birth, but they do not directly implicate a causal relationship. Future studies focusing on these specific parameters might provide a more nuanced insight into the multifactorial influences on preterm birth in the context of antiphospholipid antibody-associated thrombophilia.

In the context of our investigation into the association between aPL antibodies and preterm birth, it is essential to highlight the difference between the presence of these antibodies and the clinical diagnosis of APS. While our study found a consistent relationship between aPL antibodies and an increased risk of preterm birth, we did not specifically differentiate between the impact of clinically diagnosed APS and mere antibody positivity. The presence of aPL antibodies is a characteristic feature of APS, but the syndrome’s diagnosis also involves other clinical manifestations such as thrombosis. The comparative impact of APS versus only antibody positivity on preterm birth risk remains an area that warrants further exploration. A nuanced understanding of this relationship could yield more targeted clinical interventions and inform future research about the underlying mechanisms linking APS with adverse pregnancy outcomes. Future studies that separate these two aspects might uncover more precise information about how APS as a whole, compared to the presence of the antibodies alone, influences the risk of preterm birth.

The current systematic review provides a solid foundation for further research on the impact of aPL-associated thrombophilia on preterm birth and fetal development. These findings clearly indicate a relationship between the presence of aPL antibodies and an increased risk of preterm birth. Future studies should aim to better understand the potential underlying mechanisms, evaluate the effect of different types of aPL antibodies on preterm birth risk, and investigate potential interventions to mitigate this risk. Additionally, more research is needed to explore potential modifiers, such as maternal age, coexisting maternal conditions, and gestational age at thrombophilia diagnosis, to provide a more comprehensive understanding of the impact of aPL-associated thrombophilia on pregnancy outcomes.

### 4.2. Study Strengths and Limiations

The strengths of this systematic review lie in its comprehensive and structured approach. A rigorous search strategy was implemented, which was inclusive of four major databases, ensuring the capture of a wide range of relevant studies. The diversity of the studies, both geographically and methodologically, broadens the scope and generalizability of the review’s findings. Our review also took into account various study designs (cohort and case–control), which contributes to a more rounded picture of the impact of aPL-associated thrombophilia on preterm birth and fetal development. Further, by evaluating both study characteristics and key findings, our review provided a detailed and thorough analysis of the relationship between aPL-associated thrombophilia and preterm birth.

However, certain limitations also need to be acknowledged. Although most studies agreed on the association between aPL antibodies and preterm birth, there was considerable variability in the presence of specific aPL antibodies and the reported risk of preterm birth. Furthermore, there were inconsistencies across the studies in reporting key patient characteristics such as BMI, smoking status, and ethnicity, which could have potentially modified the observed relationship. Not all studies reported on the same risk factors or patient characteristics, which may have affected the comparison and synthesis of results. Finally, despite our thorough search strategy, our review was limited to English-language publications, which may have resulted in missing relevant studies published in other languages.

## 5. Conclusions

Based on the comprehensive analysis of the selected studies, it is evident that antiphospholipid (aPL) antibodies significantly impact preterm birth and, potentially, fetal development. The review consistently noted an association between aPL-associated thrombophilia and preterm birth, regardless of the geographical location or study design. The findings also suggest that the type and number of aPL antibodies present can influence the risk of preterm birth, highlighting the complexity of antiphospholipid antibody-associated thrombophilia in pregnancy. This systematic review underscores the necessity for thorough screening and monitoring of pregnant women with aPL antibodies to mitigate adverse fetal outcomes. Future research should aim to clarify the precise mechanisms through which aPL antibodies impact fetal development, to further refine therapeutic strategies and improve outcomes for this patient group.

## Figures and Tables

**Table 1:** Study characteristics.

Study and Author	Country	Study Year	Study Design	Study Quality
1 Saccone G et al. [21]	Italy	2017	Retrospective Cohort	Good
2 Bouvier S et al. [22]	France	2016	Prospective Cohort	Excellent
3 Alijotas-Reig J et al. [23]	Spain	2019	Prospective and Retrospective	Good
4 Rezk M et al. [24]	Egypt	2016	Prospective Cohort	Fair
5 Deguchi M et al. [25]	Japan	2017	Retrospective Cohort	Fair
6 Gabbay-Benziv R et al. [26]	Israel	2017	Retrospective Cohort	Good
7 Iordache O et al. [27]	Romania	2022	Retrospective Cohort	Good
8 Jin J et al. [28]	China	2022	Case–Control	Fair

**Table 2:** Patients’ characteristics.

Study and Author	Patients Number	Age (Years)	BMI (kg/m^2^)	Ethnicity	Smoking
1 Saccone G et al. [21]	750	27.8 ± 6.9	25.5	Caucasian: 91.9%	8.7%
2 Bouvier S et al. [22]	517	30 (16–41)	26.0	Caucasian: 94.6% African-American: 4.2% Asian: 1.2%	9.7%
3 Alijotas-Reig J et al. [23]	1000	35.2 ± 5.9	24.2	Caucasian: 72.5% African-American: 2.7% Asian: 0.5%	15.2%
4 Rezk M et al. [24]	162	31.3 ± 4.4	25.0	NR	NR
5 Deguchi M et al. [25]	81	34.1 ± 4.0	NR	NR	NR
6 Gabbay-Benziv R et al. [26]	99	33 (21–41)	23.7	NR	NR
7 Iordache O et al. [27]	211	42.9% ≥ 35	19.2% > 25	NR	15.4%
8 Jin J et al. [28]	115	32.2 ± 4.0	NR	NR	NR

NR—Not reported; BMI—Body mass index.

**Table 3:** Pregnancy and patient outcomes.

Study and Author	Previous Pregnancy History	History of Risk Factors	Presence of APL Antibodies	Preterm Birth Risk Assessment OR/RR (95% CI)
1 Saccone G et al. [21]	Livebirth: 33.9%	DM: 4.5% Thrombosis: 15.9% Fetal loss < 10 weeks: 25.3%	Single LA: 7.2% Single aCL: 61.0% Single ab2GPI: 17.1% Double positive: 12.0% Triple positive: 2.7%	Single positive vs. multiple: RR = 0.71 (0.51–0.90)
2 Bouvier S et al. [22]	Livebirth: 75.4%	Hyperlipidemia: 11.2% Thrombosis: 0.0% Fetal loss < 10 weeks: 18.5%	LA: 61.7% aCL: 47.2% ab2GPI: 22.1%	aCL: OR = 1.58 (0.95–2.64) ab2GPI: OR = 1.59 (0.79–3.22)
3 Alijotas-Reig J et al. [23]	Livebirth: 72.8%	DM: 1.8% Thrombosis: 3.1% Fetal loss < 10 weeks: 27.0%	Single LA: 35.6% Single aCL: 22.4% Single ab2GPI: 12.6% Double positive: 18.4% Triple positive: 11.0%	No treatment vs. treatment: OR = 2.44 (1.18–6.20)
4 Rezk M et al. [24]	NR	Thrombosis: 17.3% Fetal loss < 10 weeks: 49.4%	LA: 37.0% aCL: 92.6% ab2GPI: 25.3%	Triple positive: OR = 1.29 (1.09–1.53) ab2GPI vs. triple positive: RR = 0.74 (0.63–0.86)
5 Deguchi M et al. [25]	Livebirth: 82.7%	Thrombosis: 29.6% SLE: 43.2% Fetal loss < 10 weeks: 30.8%	LA: 61.7% aCL: 50.6% ab2GPI: 55.5% Double or triple positive: 54.3%	No treatment vs. treatment: OR = 8.74 (1.69–45.2) Double or triple positive: OR = 9.61 (1.78–51.8)
6 Gabbay-Benziv R et al. [26]	NR	DM: 11.1% Thrombosis: 33.3% SLE: 17.2% Fetal loss < 10 weeks: 18.2%	Single LA: 46.5% Double positive: 18.2% Triple positive: 35.4%	Single positive vs. multiple: RR = 0.70 (0.62–0.94)
7 Iordache O et al. [27]	Livebirth: 11.8%	DM: 4.7% Thrombosis: 23.7% Fetal loss < 10 weeks: 74.4%	APL antibodies: 14.7%	First trimester pregnancy loss: 4.47 (2.03–6.32) Second trimester pregnancy loss: 3.85 (1.83–5.41)
8 Jin J et al. [28]	Livebirth: 26.1%	Thrombosis: 5.1%	LA: 26.9% Double positive: 29.6% Triple positive: 11.3%	Double or triple positive: 5.40 (1.35–21.5)

APL—Antiphospholipid; LA—Lupus anticoagulant; aCL—Anticardiolipin; ab2GPI—Anti-β2 glycoprotein I antibodies; OR—Odds ratio; RR—Risk ratio; DM—Diabetes mellitus; NR—Not reported; CI—Confidence interval; SLE—Systemic lupus erythematosus.

## Data Availability

Not applicable.
